# Gut-Derived Serotonin Contributes to the Progression of Non-Alcoholic Steatohepatitis *via* the Liver HTR2A/PPARγ2 Pathway

**DOI:** 10.3389/fphar.2020.00553

**Published:** 2020-05-14

**Authors:** Lulu Wang, Xiangcheng Fan, Jichun Han, Minxuan Cai, Xiaozhong Wang, Yan Wang, Jing Shang

**Affiliations:** ^1^State Key Laboratory of Natural Medicines, China Pharmaceutical University, Nanjing, China; ^2^Division of Clinical Pharmacy, Department of Pharmacy, Drum Tower Hospital Affiliated to Medical School of Nanjing University, Nanjing, China; ^3^Jiangsu Key Laboratory of TCM Evaluation and Translational Research, China Pharmaceutical University, Nanjing, China; ^4^Department of Hepatology, Traditional Chinese Medicine Hospital, Xinjiang Uygur Autonomous Region, Urumqi, China; ^5^Department of Traditional Chinese Medicine, Chengdu Fifth People’s Hospital, Chengdu, China

**Keywords:** gut-derived serotonin, non-alcoholic fatty liver disease, non-alcoholic steatohepatitis, HTR2A, PPRAγ2

## Abstract

The precipitous increase in occurrence of non-alcoholic steatohepatitis (NASH) is a serious threat to public health worldwide. The pathogenesis of NASH has not yet been thoroughly studied. We aimed to elucidate the interplay between serotonin (5-hydroxytryptamine, 5-HT) and NASH. The serum 5-HT levels in patients with non-alcoholic fatty liver disease (NAFLD) and a rat fed with high fat-sucrose diet (HFSD) were evaluated using liquid chromatography-hybrid quadrupole time-of-flight mass spectrometry (LC-QTOF MS)/MS. The peripheral Tph1 inhibitor, LP533401, and a tryptophan (TRP)-free diet were administered to rats with NASH, induced by HFSD. BRL-3A cells were treated with 1 mM free fatty acids (FFAs) and/or 50 μM 5-HT, and then small interfering RNA (siRNA) targeting the 5-HT2A receptor (HTR2A) and the PPARγ pharmaceutical agonist, pioglitazone, were applied. We found a marked correlation between 5-HT and NASH. The absence of 5-HT, through the pharmaceutical blockade of Tph1 (LP533401) and dietary control (TRP-free diet), suppressed hepatic lipid load and the expression of inflammatory factors (*Tnfα*, *Il6*, and *Mcp-1*). In BRL-3A cells, 50 μM 5-HT induced lipid accumulation and upregulated the expression of lipogenesis-ralated genes (*Fas*, *Cd36*, and *Plin2*) and the inflammatory response. Specifically, HTR2A knockdown and evaluation of PPARγ agonist activity revealed that HTR2A promoted hepatic steatosis and inflammation by activating PPARγ2. These results suggested that duodenal 5-HT was a risk factor in the pathological progression of NASH. Correspondingly, it may represent an attractive therapeutic target for preventing the development of NASH *via* the regulation of the HTR2A/PPARγ2 signaling pathway.

## Introduction

Non-alcoholic fatty liver disease (NAFLD) is emerging as a chronic liver disease worldwide, especially in affluent areas within Western countries. It is characterized by the extensive accumulation of fats in the liver, and always associated with obesity, insulin resistance (IR), type 2 diabetes mellitus, drug-induced liver injury, and other metabolic syndrome diseases (Kawano and Cohen, 2013; Willebrords et al., 2015). Approximately 10–20% of patients with NAFLD experience non-alcoholic steatohepatitis (NASH), which may give rise to cirrhosis, hepatic fibrosis, and eventually hepatocellular carcinoma; some liver-related mortality also occurs (Loomba and Sanyal, 2013; Singh et al., 2015). The pathogenic mechanisms of NASH are usually caused by “multiple parallel hits,” which originate from the gut-liver axis and/or the adipose tissue, and include steatosis, environmental and genetic factors, oxidative and inflammation stress, and insulin resistance (Tilg and Moschen, 2010). More recently, the increase in liver- and non-liver related morbidity and mortality has been extensively reported in the world (Brodosi et al., 2017). Nevertheless, there has been no ratification of pharmacological drugs with clear therapeutic mechanisms for NASH that are able to meet the clinical demands, although various nutraceuticals and phytochemicals has been tested.

Serotonin (5-hydroxytryptamine, 5-HT) is a multi-functional bioamine with integral roles in the regulation of numerous physiological and pathological pathways. Approximately 90–95% of the total body content of 5-HT is synthesized and secreted from enterochromaffin (EC) cells, which are found in the gastrointestinal (GI) tract epithelium (Martin et al., 2017). 5-HT exerts its biological action mainly by binding 5-HT receptors (HTRs), where it is then taken up by 5-HT transporters (SERT, slc6a4) and metabolized by monoamine oxidase (MAO) (Wade et al., 1996). The action of gut-derived serotonin (GDS) and the known targets of 5-HT outside of the GI tract have garnered our attention. Intriguingly, as a gastrointestinal hormone, 5-HT is able to directly regulate the liver, thereby mediating liver regeneration (Lesurtel et al., 2006). However, the mechanism of interplay between GDS and NASH remains unclear. A greater understanding of 5-HT in the pathogenesis of NASH may provide a meaningful significant advance in the field of pharmaceutical development.

The action of 5-HT is dependent on its receptors. Early studies found 14 subtypes of HTRs; with the exception of 5-HT3R, which is a ligand-gated cation channel, all the others are G protein coupled receptors (GPCRs) (Barnes and Sharp, 1999). Recently, evidence has emerged to emphasized the involvement of 5-HT in the pathogenesis and development of NAFLD (Nocito et al., 2007; Li et al., 2018). Studies have suggested that 5-HT may raise the fat concentration of the liver (Haub et al., 2011). Moreover, a few studies have revealed that the activation of hepatic HTRs may play a pivotal role in the regulation of hepatic steatosis through the gut-liver axis (Choi et al., 2018). The fat concentration, inflammation, and cellulars necrosis in the liver of ob/ob mice were all decreased following treatment with a 5-HT receptor 3 (HTR3) antagonist; this occurred through the reduction of the elevated serotonin levels in the intestine (Haub et al., 2011). Tryptophan supplementation had no impact on the small intestinal barrier or fatty liver disease (Ritze et al., 2013). Nonetheless, although these findings described the effect of 5-HT metabolism abnormity in NAFLD, further clarification was needed determine whether GDS could directly control hepatic lipid accumulation and inflammation. In addition, the signaling pathway that mediates 5-HT-induced abnormality of hepatic lipid metabolism remains to be elucidated.

Peroxisome proliferator-activated receptor γ (PPARγ) serves as a ligand-dependent transcription factors that plays a pivotal role in the regulation of lipid utilization and storage, which are likely to contribute to the development of NASH (Yamazaki et al., 2011). In addition, hepatocyte-specific knockout PPARγ attenuated hepatic steatosis in high-fat diet (HFD)-fed mice (Morán-Salvador et al., 2011). Recent reports have indicated that HTR activation impaired insulin secretion and mitochondrial activity through stimulation of PPARγ expression (Cataldo et al., 2017). Consequently, further investigation is warranted into whether GDS regulated hepatic lipid disorder and the inflammatory response through the activation of hepatic HTRs that subsequently activate PPARγ.

To test this, we aimed to investigate what was known about the interplay between GDS and NASH. We also examined GDS in terms of the regulation of hepatic steatosis and inflammation through the HTR2A/PPARγ pathway.

## Material and Methods

### Subjects

From April 2016 to June 2017, 200 subjects in total were examined at the Physical Examination Center of Xinjiang Traditional Chinese Medicine Hospital. Overall, 100 cases of NAFLD were detected; the patients comprised 65 men and 35 women, between 23 and 82 years of age, inclusive (48.04 ± 14.16 years of age). There were 100 healthy subjects, 41 males and 59 females, aged 21–60 years (37.05 ± 10.62 years). In this clinical trial, the informed consent was obtained from all subjects, and this project was approved by the ethics committee as following attachment. The diagnostic criteria for NAFLD are based on the 2010 Chinese Medical Association Liver Diseases Committee “Guidelines for the diagnosis and treatment of NAFLD (modified version)” (Cao and Fan, 2011). All subjects were diagnosed or excluded from fatty liver by fasting B-ultrasound diagnosis and semi-quantitative analysis (Ballestri et al., 2015; Ferraioli and Monteiro, 2019)

The criteria for patients with NAFLD required an absence of drinking history or regular drinking of < 140 g per week for men and < 70 g per week for women, and excluded patients with viral hepatitis, drug-induced hepatitis fatty liver caused by specific diseases such as hepatolenticular degeneration, total parenteral nutrition, and autoimmune liver disease.

### Animals and Treatments

All animal experiments conformed to the relevant animal experimentation ethics regulations. The animal experiments protocols of this study were approved by the Animal Experimentation Ethics Committee of China Pharmaceutical University [Approval ID: SCXK (Hu) 2013–0016]. Forty-eight male Sprague-Dawley rats (body weight 180–200 g) were purchased from Shanghai Super-B&K Laboratory Animal Corp. Ltd. (Shanghai, China). All rats were kept in a controlled environment (12 h light/dark cycle), with *ad libitum* access to food and water. For the rats first week of acclimatization, rats were fed standard chow.

After acclimatization, these animals were divided into eight groups (n=6 per group, **Figure 2A**) based on body weight. Control groups: The rats fed basal diet (360 kcal/100 g, 13.3 g/100 g from fat, 26.2 g/100 g from protein, and 60.5 g/100 g from carbohydrate) and sacrificed at 20 days (C20) and at 30 days (C30), Model groups: The rats fed high fat-sucrose diet (506.8 kcal/100 g, lard, 10 g/100 g; cholesterol, 2 g/100 g; egg yolk power, 5 g/100 g; sucrose, 10 g/100 g; propylthiouracil, 2 g/100 g; basal diet, 72.8 g/100 g) and sacrificed at 20 days (M20) and at 30 days (M30), tryptophan (TRP)-free diet groups: The rats fed high fat-sucrose diet without tryptophan, and sacrificed at 20 days (R20) and at 30 days (R30), Tph1 inhibitor groups: The rats fed high fat-sucrose diet with LP533401 treatment (LP533401:17.5 mg·kg^-1^·day^-1^ by gavage, Dalton Pharma Services, Toronto, Ontario, Canada), and sacrificed at 20 days (T20: treated with LP533401 from day 0 to day 20) and at 30 days (T30: treated with LP533401 from day 20 to day 30). All feed was provided by Trophic Animal Feed High-tech Co. Ltd (Nantong, China) (Wang et al., 2017). On days 20 and 30, the rats were killed by pentobarbital sodium (Merck, Germany) anesthesia following a 12 h fast in accordance with the experimental protocols. Blood and liver samples were then collected for further analyses.

### Cell Culture

The BRL-3A cells were purchased from the Cell Bank of Type Culture Collection of the Chinese Academy of Sciences (Shanghai, China). All cells were cultured in Dulbecco’s modified Eagle medium (DMEM) (HyClone, Logan, UT, USA) supplemented with 10% (v/v) FBS, 100 U·ml^−1^ penicillin and 100 μg·ml^−1^ streptomycin (Gibco, Grand Island, NY) at 37°C in a humidified atmosphere with 5% CO_2_. Cell steatosis was induced by the addition of 1mM free fatty acids (FFAs, OA: PA, 2:1, Sigma-Aldrich) for 24 h, and FFA-free bovine serum albumin was added at an equal final concentration to the control cells. When the cells reached a confluence of 60–70%, they were plated in 6-well plates (1×10^6^ cells/well) with or without the fatty acid mixture, 5-HT, and other drugs in serum-free DMEM. Specific groups, comprising: (a) the control group (1% bovine serum albumin (BSA) with free FFAs), (b) the model group (1 mM FFAs with 1% BSA), (c) the 5-HT group (treated with 50 μM 5-HT (Sigma-Aldrich) only), and (d) the 5-HT+FFA group (treated with FFAs and 50 μM 5-HT). For further target and signal verification, the cells were treated with TCB-2 (0.1 μM, APExBIO Technology), ketanserin (1 μM, Sigma-Aldrich), pioglitazone (10 μM, Aladdin), GW9662 (1 μM, APExBIO Technology). After the cells were incubated in different medium for 24 h, the hepatocellular lipid accumulation was evaluated by triglyceride measurement and oil red O staining.

### Cell Transfection

HTR2A small interfering RNA (siRNA) sequences, control siRNA sequences, and siRNA transfection reagent (Lipofectamine 2000) were purchased from Shanghai GenePharma Co., Ltd (Shanghai, China). In accordance with the manufacturer’s protocol, 100 nM siRNA was transfected into BRL-3A cells for 6 h. Thereafter, the cells were switched into fresh medium for a further 24 h. Subsequently, the cells were exposed to the test treatments.

### Biochemical Analyses

Blood samples were obtained from ophthalmic vein of rats under anesthesia and immediately centrifuged at 1,200 g for 15 min. The serum levels of total cholesterol (TC), triglyceride (TG), LDL, HDL, alanine aminotransferase (ALT), and aspartate aminotransferase (AST) were evaluated by using a biochemical analysis kit (Jiancheng Bioengineering Institute, Nanjing, China). Serum TNF-α, interleukin (IL)-1β, and IL-6 were examined by using ELISA kits from Neobioscience (Neobioscience Technology Co., Ltd., Shenzhen, China).

### Quantification of Serotonin

Blood samples from the human study subjects and rats were centrifuged twice ensure the supernatant was obtained with no platelets. The initial force was 700 g for 10 min, followed by 1,200 g for 15 min. For gut tissue collection, 2–3 cm sections of each intestinal tissues were cut and stained with 5-HT antibody (1:10,000, ImmunoStar) for immunohistochemical analysis. Sections of approximately 5 cm of the remaining gut and liver samples were collected and prepared for subsequent 5-HT content analysis by LC-MS (**Tables S1** and **S2**).

### Histological Analysis

The liver tissues were fixed in 4% (w/v) paraformaldehyde solution for 24 h, dehydrated in a gradient series of ethanol, and embedded in paraffin wax. Slices of 4 μm were cut and stained with H&E and Sirius red. The NAFLD activity score (NAS) was evaluated in a blinded manner by a professional pathologist in accordance with the criteria described previously by Brunt et al. (Kleiner et al., 2005). To visualize the accumulation of hepatic fat droplets, fresh frozen liver samples were embedded in optimal cutting temperature (OCT)-freeze medium and sliced into 4 μm sections. The frozen sections were stained with oil red O (Sigma Aldrich) for 30 min, counter-stained with H&E for 30 s, then mounted in neutral resin. The slides were observed by using an optical light microscope (Olympus-BX53, Tokyo, Japan). The contents of liver lipid droplets were normalized and calculated by using Image-J software for semiquantitative analysis. For histological analysis, macrophages were stained with class D scavenger receptor CD68-positive antibody (1:800, Abcam, Cambridge, UK).

### Quantitative Real-Time Polymerase Chain Reaction

Total tissue RNA was isolated from the rat jejunum, ileum, and colon fractions by using TRIzol reagent (Invitrogen, Carlsbad, CA, USA) and reverse transcribed into complementary DNA (cDNA) by using PrimeScript™ RT Master Mix (Takara, Japan) in accordance with the manufacturer’s instructions. The coding sequence of the receptor, as published in GeneBank, was used to design specific oligonucleotide primers (Generay Biotech Co., Shanghai, China) for the purpose of gene sequencing (**Table S3**). Gene expression analysis was performed by using StepOnePlus™ real-time PCR sequence detection system (Applied Biosystems, USA) with power SYBR^®^ Premix Ex TaqTM (Takara, Kyoto, Japan). Gene expression in each group was normalized. Glyceraldehyde-3-phosphate dehydrogenase (GAPDH) was used as an internal reference during parallel amplification. The relative quantification of gene expression was performed by using the 2^−ΔΔt^ calculation method. All experimental procedures for reverse transcription quantitative PCR from primer design to the relative quantitative of the gene sequence and the environment and methods of detection were in accordance with Minimum Information for Publication of Quantitative Real-Time PCR Experiments (MIQE) guidelines (Bustin et al., 2009).

### Western Blotting Analysis

The protein content of the rat liver tissue sections and the lysed cells were homogenized in an extraction buffer at 4°C, and extracted using a protein extraction kit (Pulilai Gene Technology Co., Ltd., Beijing, China). The protein was diluted and quantified using a bicinchoninic acid kit (Jiancheng Bioengineering Institute, Nanjing, China), then mixed with prototype 5× sodium dodecyl sulfate polyacrylamide gel electrophoresis (SDS-PAGE) loading buffer. The samples were denatured of mixture at 95°C for 5 min in a metal bath and then resolved by electrophoresis on a 6–10% polyacrylamide gel. The separated proteins were transferred onto nitro-cellulose membranes and incubated of the blots with 5% BSA blocking buffer for 1.5 h. Subsequently, the protein bands were detected by incubation with specific primary antibodies [HTR2A, PPARγ2, and HTR2B (1:1,000, Santa Cruz), HTR2C (1:1,000, Abcam), and β-actin (1:2,000, Sigma-Aldrich)] in Tris buffered saline buffer with Tween 20 (TBST) overnight at −4°C. The protein bands were washed three times for 10 min each, and then incubated with secondary antibodies [goat anti-rabbit immunoglobulin G (IgG), HRP (1:8,000, Jackson ImmunoResearch) and goat anti-mouse IgG, HRP (1:4,000, Abcam)] for 1–2 h at 25°C. Unbound antibodies were removed by washing in TBST, and protein-antibody linked bands were viewed by the application of enhanced chemiluminescence reagents (Millipore, USA). The expression of each protein band was normalized to the expression of β-actin, the internal control. The optical density of each band was scanned and semi-quantified by using Quantity One software (Bio-Rad, Hercules, CA, USA)

### Statistics

The data from each group are presented as the mean ± standard error of the mean (SEM). Multiple comparisons were made using the one-way analysis of variance (ANOVA) and Student’s *t*-test for unpaired samples. These statistical analyses were computed by using GraphPad Prism version 5.01 (GraphPad Software, San Diego, California, CA, USA). P values less than 0.05 (P < 0.05) indicated statistically significant differences.

## Results

### Increased Gut-Derived Serotonin Signaling in the Duodenum Was Correlated With Non-Alcoholic Fatty Liver Disease/Non-Alcoholic Steatohepatitis

**Figure 1:**
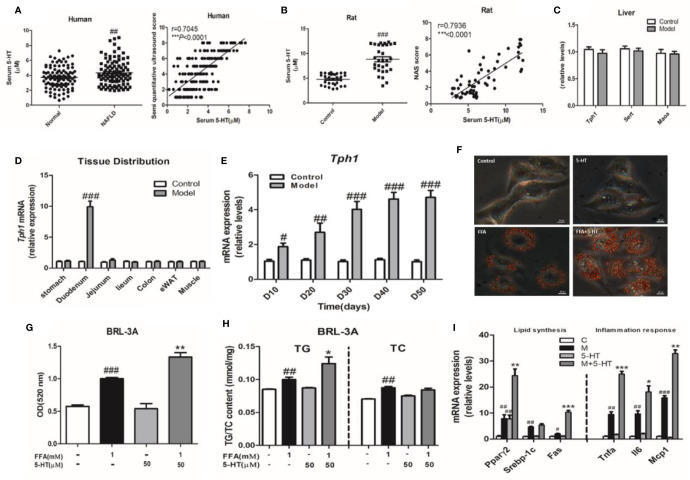
Increased gut-derived serotonin (GDS) signaling in the duodenum was correlated with non-alcoholic fatty liver disease (NAFLD)/non-alcoholic steatohepatitis (NASH). **(A)** The level of serum 5-hydroxytryptamine (5-HT) and correlation analysis between serum 5-HT and semi-quantitative ultrasound score in healthy humans and patients with NAFLD (n = 100), as well as in the control and model groups (NASH SD rats) (n = 30) **(B)**. **(C)** The messenger RNA (mRNA) expression of *Tph1*, *Sert*, and *Maoα* in the liver of SD rats. **(D)** The tissue distribution of *Tph1* mRNA expression in SD rats. **(E)** The duodenal *Tph1* mRNA expression in SD rats fed an high fat-sucrose diet (HFSD) diet for 10, 20, 30, 40, and 50 days. **(F)** Oil red O staining of BRL-3A (× 400). Small red circles indicate the formation of large cytoplasmic lipid droplets. **(G)** The lipid content in BRL-3A. **(H)** The content of triglyceride (TG) and total cholesterol (TC) in BRL-3A. **(I)** The expression of genes related to lipid synthesis (*Pparγ2*, *Srebp1c*, and *Fas*) and the inflammatory response (*Tnfα*, *Il6*, and *Mcp1*) in BRL-3A cells. All data are presented as the mean ± SEM (n = 6). ^#^*P* < 0.05, ^##^*P* < 0.01, and ^###^*P* < 0.001, compared with the corresponding control group; *P < 0.05, ^**^P < 0.01 and ^***^P < 0.001 compared with the model group.

In total, 200 subjects with or without NAFLD were evaluated during the study period. Of these, 100 subjects were confirmed to have NAFLD by B-ultrasound. Of these, we first investigated whether 5-HT was involved in the progression NAFLD patients. We observed a marked upregulation in serum 5-HT (**Figure 1A**). The serum quantitative ultrasound score showed an excellent correlation with serum 5-HT (r=0.7045, p < 0.0001). Therefore, we speculated that serum 5-HT may have been associated with NAFLD. In addition, the serum and duodenum of 5-HT was also upregulated in rats with HFSD-induced NASH (**Figures 1B** and **S1A–C**). The serum 5-HT level was positively correlated with the NAFLD activity score (NAS) (r=0.7936, p < 0.0001) (**Figure 1B**). Next, we determined whether the liver could produce 5-HT and therefore be involved in 5-HT metabolism. However, gene expression of neither *Tph1* nor *Sert* and *Maoα* was significantly different in the liver of rats with NASH compared with rats fed an standard chow diet (SCD) (**Figure 1C**). As almost all the 5-HT in our body is synthesized in the periphery, especially in the GI mucosa. Therefore, we examined the expression of the *Tph1* gene in different tissues. Notably, *Tph1* expression was increased in the duodenum and was clearly different during the progression of NASH compared with rats fed an SCD (**Figures 1D, E**). To determine whether 5-HT accelerated lipid accumulation *in vitro*, we followed the change in hepatocellular lipid deposition induced by FFAs with 5-HT treatment in BRL-3A cells. Our results showed that 5-HT with FFA substantially increased lipid droplet accumulation and intracellular TG, but without alteration in the TC content (**Figures 1F–H**). Both lipogenesis-related genes *(Pparγ2* and *Fas*) and inflammation-related genes (*Tnfα*, *Il6*, and *Mcp1*) were significantly increased in the FFA-treated group. However, 5-HT increased *Fas*, *Pparγ2*, *Tnfα*, *Il6*, and *Mcp1* messenger RNA (mRNA) expression when added to FFA treatment with no alteration of *Srebp1c* (**Figure 1I**). Overall, these data indicated that GDS was involved and precipitated the development of NAFLD/NASH.

### Gut-Derived Serotonin Deficiency Ameliorated the Progression of Non-Alcoholic Steatohepatitis

**Figure 2:**
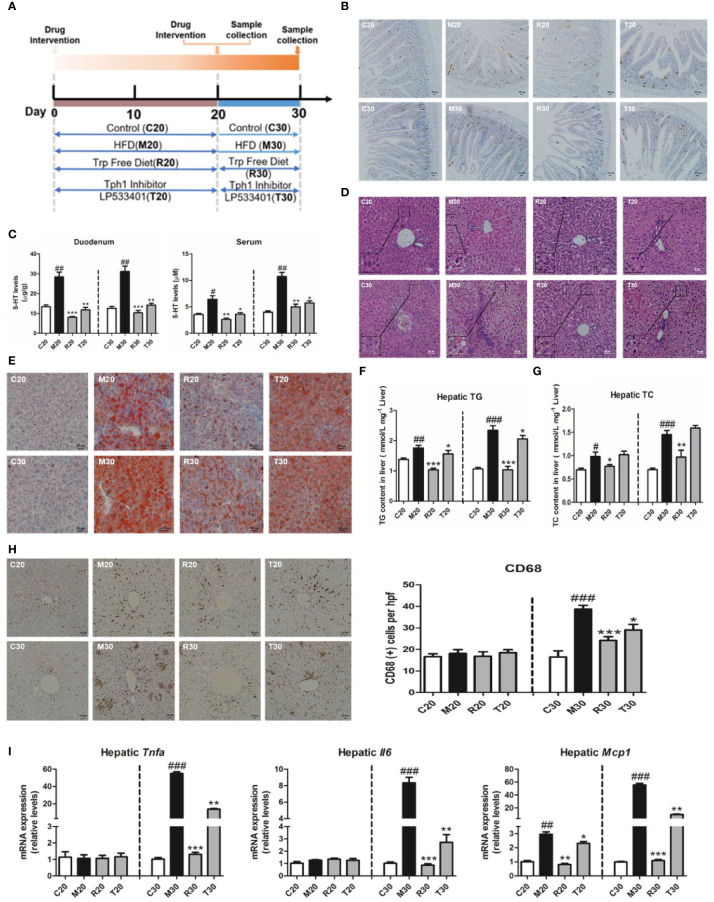
Gut-derived serotonin (GDS) deficiency ameliorated the progression of non-alcoholic steatohepatitis (NASH). **(A)** Experimental procedure. **(B)** The content of 5-hydroxytryptamine (5-HT) positive cell in duodenum of SD rats. **(C)** The content of 5-HT in serum and duodenum of SD rats. **(D)** Hematoxylin–eosin (H&E) staining results of each group of rats (evaluated by 6 experts, ×200). **(E)** Oil red O staining (small red circle) shows hepatic lipid deposits (×200). Hepatic TG concentrations **(F)** and TC concentrations **(G)** in SD rats. **(H)** The CD68 positive cells content (×100). **(I)** The messenger RNA (mRNA) expression of genes related to the inflammatory response (*Tnfα*, *Il6*, *Mcp1*) in SD rats fed an HFSD diet on days 20 and 30. The terms C20, C30, and M20, etc., are explained in *Animals and Treatments*. All data are presented as the mean ± SEM (n = 6). ^##^*P* < 0.01, ^###^*P* < 0.001, compared with corresponding control group; ^*^P < 0.05, ^**^P < 0.01, ^***^P < 0.001 compared with relative model group.

Our previous report showed the HFSD induced NASH in rats at day 30 and non-alcoholic fatty liver (NAFL) condition at day 20 (Wang et al., 2017). To further explore whether the depletion of 5-HT could alleviate the pathogenesis of NASH, we applied LP533401 and tryptophan-free diet in this study (**Figure 2A**). As our results showed, the levels of TG and TC were markedly reduced in rats fed LP533401 and a TRP-free diet on days 20 and 30 compared with those in SCD-fed rats (**Figures S2A, B**). Moreover, it also lowered the LDL level and elevated the level of HDL level in the TRP-free diet group (**Figures S2C, D**). Elevated serum ALT and AST, typical characteristics of NASH, are reflective of hepatocellular injury. Notably, the TRP-free diet with LP533401 caused decreased AST and ALT levels on day 30 (**Figures S2E, F**); this diet with LP533401 treatment also significantly decreased the liver weight and body weight of the rats compared to HFSD (**Figures S2H, I**), but without any change in food intake (**Figure S2G**).

In addition to the 5-HT produced within the central nervous system, evidence has emerged to indicate that peripheral 5-HT is a factor that promotes nutrient absorption and storage. We found that the 5-HT levels in the liver, duodenum, and serum of 5-HT were upregulated on days 20 and 30, whereas rats fed the TRP-free diet and treated with LP533401 had markedly reduced 5-HT levels (**Figures 2B, C**, and **S3A**). We also observed a clear reduction in lipid droplets (**Figures 2E** and **S3C**) and hepatic TG and TC levels (**Figures 2F, G**). In control group, the distribution of CD68-positive KCs was very sporadic. After the group was fed a TRP-free diet and treated with LP533401, a lower level of CD68-positive KCs was observed (**Figure 2H**). Consistent with the results of H&E staining (**Figure 2D**), the hepatic expression of inflammation-related mRNA was dramatically downregulated on day 30 (**Figures 2I** and **S3D**) and NAS was also reduced (**Figure S3B**). However, only hepatic *Mcp1* expression was downregulated on day 20, with no significant differences in *Tnfα* and *Il6*. The above results further suggested that the abolition of serotonin had an important role against HFSD-induced NASH.

### Gut-Derived Serotonin Promoted Lipid Synthesis and the Inflammatory Response Through the HTR2A Receptor in Hepatocytes

**Figure 3:**
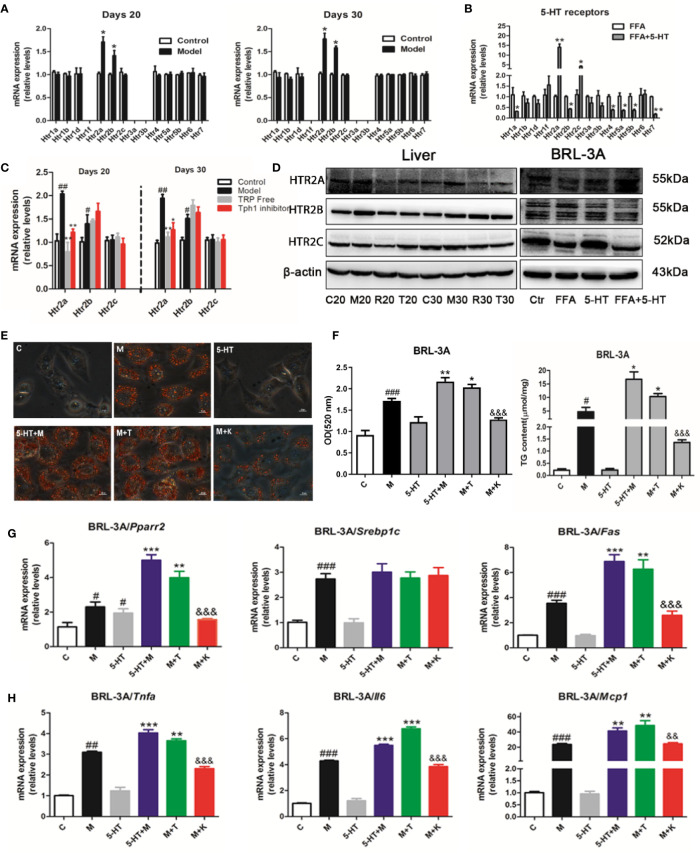
Gut-derived serotonin (GDS) promoted lipid synthesis and the inflammatory response through the HTR2A receptor in hepatocytes. **(A)** Relative messenger RNA (mRNA) expression of 14 HTRs on day 20 and day 30 in SD rats. **(B)** Relative mRNA expression of 14 HTRs in BRL-3A. **(C)** Relative gene expression of *Htr2a*, *Htr2b*, *Htr2c* in SD rats administrated TRP Free diet and LP5333401. **(D)** The protein level of HTR2A, HTR2B, HTR2C in SD rats fed HFSD diet for 20 or 30 days and in BRL-3A treated by free fatty acid (FFA) or 5-HT. **(E)** Oil red O staining and lipid content in BRL-3A (× 400). **(F)** The content of TG in BRL-3A. **(G)** The mRNA expression of genes related with lipid synthesis (*Pparγ2*, *Srebp1c*, *Fas*) in BRL-3A. **(H)** The mRNA expression of genes related with inflammation response (*Tnfα*, *Il6*, *Mcp1*) in BRL-3A. “C” means control group (1% BSA with FFA free), “M” means 1 mM FFA treatment; “5-HT” means 5-HT (50 μM) treatment; “5-HT + M” means 5-HT (50 μM) with FFA (1 mM) treatment; “M+T” represents FFA (1 mM) with TCB-2 (0.1 μM) treatment; “M+K” represents FFA (1 mM) and 5-HT (50 μM) with ketanserin (1 μM); All data are presented as the mean ± SEM (n = 6). ^#^*P* < 0.05, ^##^*P* < 0.01, ^###^*P* < 0.001, compared with corresponding control group; ^*^P < 0.05, ^**^P < 0.01, and ^***^P < 0.001, compared with relative model group; ^&&^*P* < 0.01 and ^&&&^*P* < 0.001, compared with “5-HT+M” group.

The most well-characterized attribute of 5-HT is the targeting of individual HTRs, which includes seven families (named 5-HT1–7) and 14 subtypes. To explore the potential mechanism through which 5-HT receptors mediated the exacerbation of NASH by 5-HT in lipid synthesis and inflammation, we examined 14 receptors in the hepatic tissue and BRL-3A cells. We found that *Htr2a* and *Htr2b* were mainly expressed in the liver on days 20 and 30 (**Figure 3A**). Similarly, the 5-HT with FFA treatment group showed upregulated *Htr2a* and *Htr2c* gene expression in BRL-3A cells compared with the control group (**Figure 3B**). The administration of LP533401 to rats fed a TRP-free diet decreased *Htr2a* mRNA and protein expression compared with the HFSD group (**Figures 3C, D** and **S4**). In contrast, 5-HT treatment of FFA group increased the protein expression of HTR2A.

To clarify whether 5-HT regulated hepatocellular lipogenesis and the inflammatory response through the HTR2A receptor, we used an HTR2A agonist (TCB-2) and an antagonist (ketanserin). Ketanserin inhibited hepatocellular neutral lipid and TG levels in FFA and ketanserin group, compared with those of the FFA-treated group in BRL-3A cells. In contrast, TCB-2 increased hepatocellular neutral lipid and TG levels (**Figures 3E, F**). Consistently, ketanserin significantly reduced *Pparγ2* and *Fas* gene expression compared with the combined FFA and 5-HT treatment group in BRL-3A cells. In addition, unlike ketanserin, TCB-2 upregulated *Tnfα*, *Il6*, and *Mcp1* mRNA expression in in FFA and TCB group, compared with that in the FFA group (**Figures 3G, H**). Then, ketanserin significantly decreased HTR2A protein levels compared with the FFA group in BRL-3A cells (**Figure 3D**). These data indicated that the activation of HTR2A promoted lipid synthesis and the inflammatory response during the progression of NASH.

### HTR2A/PPARγ2 Signaling Pathway Was Involved in the Lipogenesis and Increased the Genes Expression of Inflammation

**Figure 4:**
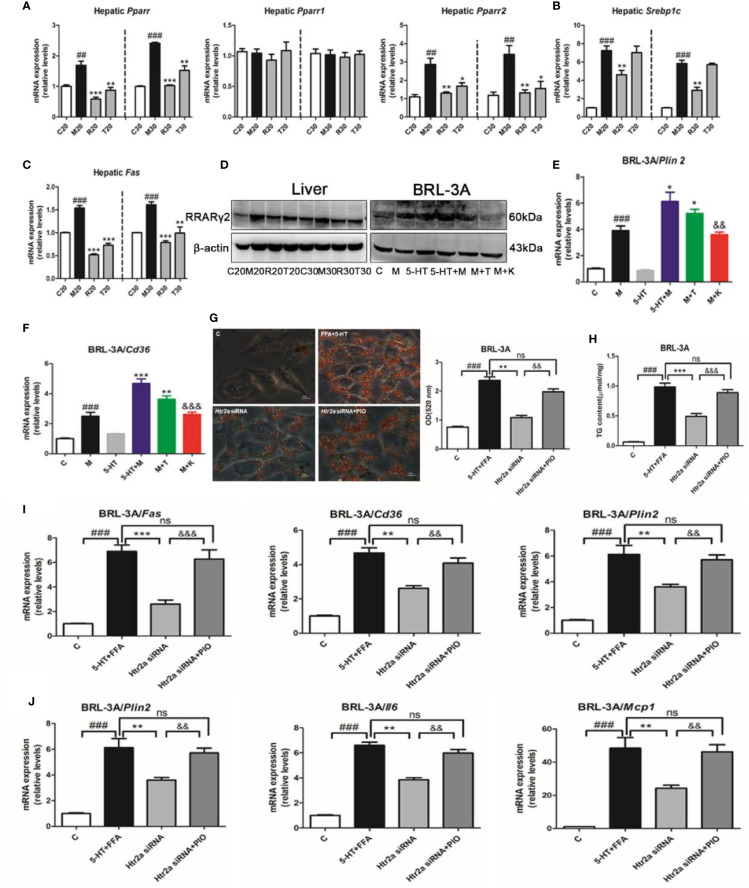
The HTR2A/PPARγ2 signaling pathway was involved in lipogenesis and the increased expression of inflammatory gene. **(A)** The messenger RNA (mRNA) expression of *Pparγ*, *Pparγ1*, and *Pparγ2* in the liver of SD rats. **(B)** The mRNA expression of *Srebp1c* in the liver of SD rats. **(C)** The mRNA expression of *Fas* in liver of SD rats **(D)** The protein expression of PPARγ2 in BRL-3A and liver of SD rats. The mRNA expression of *Plin2* **(E)** and *Cd36* **(F)** in BRL-3A. **(G)** Oil red O staining and lipid content in BRL-3A cells (×400 magnification). **(H)** The content of triglyceride (TG) in BRL-3A. **(I)** The mRNA expression of genes related to lipogenesis (*Fas*, *Cd36*, and *Plin2*) in BRL-3A. **(J)** The mRNA expression of genes related to the inflammatory response (*Tnfα*, *Il6*, and *Mcp1*) in BRL-3A cells. All data are presented as the mean ± SEM (n = 6). ^##^*P* < 0.01 and ^###^*P* < 0.001, compared with the corresponding control group; ^*^P < 0.05, ^**^P < 0.01, and ^***^P < 0.001, compared with the corresponding model group; ^&&^*P* < 0.01 and ^&&&^*P* < 0.001, compared with the 5-HT+M group. ns, represent no significance.

To further investigate the molecular pathways downstream of the HTR2A receptor that regulated hepatocellular lipogenesis and the inflammatory response, we examined the expression of PPARγ, SREBP-1c, and other lipogenesis-related genes. First, we found that HFSD significantly increased *Pparγ* and *Pparγ2* mRNA expression without alteration of *Pparγ1* compared with the SCD group, and that the administration of LP533401 to rats fed a TRP-free diet decreased hepatic *Pparγ* and *Pparγ2* gene expression compared with the HFSD group (**Figure 4A**). Compared with the HFSD group, the TRP-free diet decreased hepatic *Srebp1c* and *Fas* expression. Unexpectedly, LP533401 did not decrease hepatic *Srebp1c* mRNA expression, but downregulated hepatic *Fas* mRNA expression (**Figures 4B, C**). Also, we found that the TRP-free diet and LP533401 significantly decreased PPARγ2 protein expression in liver. Moreover, ketanserin clearly reduced PPARγ2 protein expression in BRL-3A cells (**Figures 4D** and **S5E, F**). In contrast, TCB-2 showed opposite results. Furthermore, the downstream target genes of *Pparγ2* supported this phenomenon, such as *Cd36* and perilipin 2 (*Plin2*). Ketanserin reduced gene expression of *Cd36* and *Plin2* compared with the combined treatment of FFA and 5-HT (**Figures 4E, F**).

Next, to further identify whether PPARγ directly influenced the response to lipid absorption induced by 5-HT *in vitro*. We used a PPARγ agonist (pioglitazone) and antagonist (GW9662). Unexpectedly, pioglitazone increased hepatocellular lipid droplet accumulation and TG levels compared with the FFA group in BRL-3A cells, which was consistent with 5-HT and FFA treatment (**Figures S5A, B**). In contrast with this, GW9662 reduced hepatocellular lipid levels and TG levels compared with the FFA and 5-HT-treated group in BRL-3A cells. Moreover, the downstream target genes of *Pparγ2* (*Fas*, *Cd36*, *Plin2*, *Tnfα*, *Il6*, and *Mcp1*) were all clearly elevated by pioglitazone as compared with the FFA group in BRL-3A cells (**Figures S5C, D**). In contrast, the above downstream target genes of *Pparγ2* were all substantially reduced by GW9662 compared with the combination FFA and 5-HT-treatment group. In particular, we suppressed *Htr2a* expression by using gene silencing and, simultaneously, the addition of the PPARγ agonist (pioglitazone). As expected, the *Htr2a* siRNA-treatment group strongly decreased lipid accumulation compared with the control siRNA cells (**Figure 4G**). Consistently, the TG level was also significantly decreased in BRL-3A cells treated with *Htr2a* siRNA (**Figure 4H**). Further, *Htr2a* siRNA treatment resulted in a reduction in the expression of genes related to lipogenesis and the inflammatory response (**Figures 4I, J**). Conversely, compared with the *Htr2a* siRNA-treated group, pioglitazone generally increased lipid accumulation and TG levels, and upregulated the expression of genes related to lipogenesis and the inflammatory response (**Figures 4G–J**). These results implied that PPARγ2 may serve as a sensor that mediates the promotion of HTR2A in hepatocyte steatosis and inflammation.

## Discussion

This study first explored the interplay between 5-HT and the pathological progression of NAFLD and NASH. To achieve this, we used an HFSD, as a model of a Western diet, which induced a strong increased in GDS, especially derived from the duodenum. This was in accordance with the increase in duodenal 5-HT synthesis observed in mice fed a Western diet (Bertrand et al., 2011; Bertrand et al., 2012). In parallel, we also found a positively relationship between 5-HT and patients with NAFLD. Previous studies have shown that peripheral 5-HT was associated with obesity in HFSD fed mice (Kim et al., 2011). In addition, Tph1-deficient mice fed an HFSD were protected from NAFLD by the promotion of brown adipose tissue (BAT) thermogenesis (Crane et al., 2015). The absence of GDS can alleviate and prevent the pathogenesis of NASH. Our results indicated that the abolition of 5-HT by treatment with LP533401 and TRP-free diet can alleviate the development of NAFLD/NASH. Recent studies have suggested that the abnormalities of 5-HT are closely related to NASH, but did not clarify how GDS would alter liver lipid content and inflammation.

It is known that 5-HT is synthesized from L-tryptophan by two catalysts, including tryptophan hydroxylase (Tph) and aromatic amino acid decarboxylase (AADC) (Lesurtel et al., 2008). There are two isoforms of Tph: Tph1, the rate-limiting step in the synthesis of peripheral 5-HT; and Tph2, which is predominantly expressed in the central nervous system (CNS) (Yabut et al., 2019). As 5-HT cannot pass the blood-brain barrier, virtually almost all the peripheral 5-HT originates from the enterochromaffin cells in the gut, and is then stored and released from platelets (Oh et al., 2015). Studies have previously evaluated the critically important roles of 5-HT in the regulation of energy homeostasis, through specific metabolic tissue sites, including the pancreas, adipose, bone, gut, and liver (Oh et al., 2015; Martin et al., 2017). Our data supported this, and increased serum levels of 5-HT were found in patients with NAFLD and rats with NASH. Importantly, the levels of 5-HT could potentially promote the expression of genes related to lipogenesis and the inflammatory response. In subsequent investigations, we induced GDS deficiency in rats with NASH by using the specific Tph1 inhibitor, LP533401, in the gut and by a tryptophan-free diet in general, which significantly ameliorated lipid accumulation and the inflammatory microenvironment. Recent studies established that inhibition of gut-derived Tph1 with the administration of LP533401 effectively protected against irritable bowel syndrome (Liu et al., 2008) and osteoporosis (Yadav et al., 2010) without affecting brain 5-HT content. A tryptophan-free diet is a physiological tool used to examine peripheral and brain 5-HT function (Sánchez et al., 2014). Although some studies have shown that 5-HT degradation caused oxidative stress, inflammation, and hepatocellular injury in mice with NASH induced by a choline-methionine–deficient (MCD) diet, which can be reversed in Tph1^−/−^ mice (Nocito et al., 2007). Nonetheless, no evidence has directly revealed the relationship between GDS and NASH. Our results showed that HFSD induced duodenal 5-HT rather than in the jejunum, ileum, and colon. Administration of LP533401 with a TRP-free diet reduced the 5-HT level in the duodenum, serum, and liver. These data directly suggested that GDS affected positively on NASH control and driven us to explore which pathway involved in the pathological mechanism of NASH.

In general, 5-HT exerts its biological function by binding to 5-HT receptor (HTRs) after been released. More than 14 HTRs have been identified and, with the exception of HTR3, which is a ligand-gated cation channel (Barnes and Sharp, 1999; Oh et al., 2015), they are G protein coupled receptor (GPCRs). Previous reports have shown that intestinal HTR3a and SERT were involved in the development of NAFLD in mice (Haub et al., 2010; Haub et al., 2011; Choi et al., 2018). Our results indicated that the mRNA expression of duodenal *Htr3a* and *Sert* was not change. GDS directly promoted the development and progression of NASH by hepatic HTR2A, rather than HTR2B or HTR2C, those similar to hepatic steatosis was ameliorated with liver-specific *Htr2a* knockout and the deletion of GDS (Choi et al., 2018). Similarly, the upregulation of synthesis of hepatic *Htr2a*, *Htr2b*, and 5-HT by HFSD is crucial for the HFSD-induced overproduction of hepatic triglycerides with 5-HT of administration (Li et al., 2018). These observed discrepancies may be attributable to species differences, different models used, and different administration procedures. Accordingly, it would be of interest to investigate further the role of HTR2A in the progression of NAFLD.

Surely, to further clarify the signal of HTR2A, our findings also highlighted the functional roles of PPARγ in NAFLD progression. PPARs are regarded as a ligand-activated transcription factors, which regulates numerous cell differentiation and system energy homeostasis (Gavrilova et al., 2003). The PPAR superfamily consists of three isoforms: PPARα, PPARβ/δ, and PPARγ. Of these, PPARγ, including three subtypes PPARγ1, PPARγ2, and PPARγ3, has attracted attention for its clinical and nutritional roles in the regulation of lipid and glucose metabolism (Grygiel-Górniak, 2014). Identical proteins are produced from *Pparγ1* and *Pparγ2* mRNA expression. Previous reports revealed that increasing PPARγ was always related with steatotic liver and upregulated lipogenesis through the activation of *de novo* lipogenic genes (Gavrilova et al., 2003; Derosa et al., 2018). Consistently, our results showed that 5-HT promoted hepatic *Pparγ2* expression with no change in *Pparγ1* and *Srebp1c*. Specifically, *Htr2a* knockdown by siRNA diminished lipid accumulation in BRL-3A cells. Meanwhile, after treatment with the pharmacological agonist pioglitazone, the lipid content, lipogenesis, and the expression of inflammation-related were blocked in *Htr2a* knockdown cells. Collectively, PPARγ2 is required for the activation of HTR2A-induced lipid metabolism disorder in the liver tissue.

However, there are some limitations needed to further explore in our experiment. Increasing BMI, insulin resistance, liver injury (such as AST, ALT), and hepatic lipid accumulation level (such as TG, TC) in NAFLD/NASH patients, it will offer more evidence to make clear which is the most import factor with 5-HT. Ultrasound examination has high sensitivity and specificity for tissue density, and is widely used in fatty liver screening and diagnosis (Ballestri et al., 2015; Ferraioli and Monteiro, 2019). However, ultrasound is only used as a technical means for detection and screening and preliminary diagnosis, and is not a gold standard for the diagnosis of NAFLD. Therefore, in this section, we use ultrasound for diagnosis and semi-quantitative analysis of the extent of NAFLD (absent: score 0–2, moderate: score 3–5, severe: score 6–8). Such restrictions may affect the results of the correlation analysis between serotonin in patients with NAFLD and ultrasound semi-quantitative analysis, leading to a lower correlation coefficient than that in rats.

In conclusion, our current findings have demonstrated that circulating levels of 5-HT were positively correlated with in patients with NAFLD and rats with NASH. We found that physiological or chemical inhibition of Tph1 (LP533401) protected against and/or ameliorated the progression of HFSD-induced NASH *via* activation of the HTR2A/PPARγ2 pathway. Herein, these results further suggested that blocking duodenal 5-HT synthesis may be a novel target for therapies of NAFLD/NASH.

## Data Availability Statement

The raw data supporting the conclusions of this study are available on request to the senior author (JS, shangjing21cn@cpu.edu.cn).

## Ethics Statement

The studies involving human participants were reviewed and approved by Ethics Committee of Traditional Chinese Medicine Hospital of Xinjiang Uygur Autonomous Region (2016XE0127).The animal study was reviewed and approved by Animal Experimentation Ethics Committee of China Pharmaceutical University (Approval ID: SCXK (Hu) 2013-0016).

## Author Contributions

LW designed this project and wrote the manuscript. LW and XF performed the experiments. MC drew the graphical abstract. LW, XF, and JH analyzed the data and contributed materials/analysis tools. XW and YW contributed to clinical data acquisition and methodology analysis. JS contributed to obtaining funding. JS and YW conceived and instructed the study, and critical revision of the manuscript. All these authors have critically revised the manuscript and approved the finally content.

## Funding

This work was supported by Prospective joint research project of Jiangsu Province (BY2016078-02); One Hundred Person Project of The Chinese Academy of Sciences; Applied Basic Research Programs of Qinghai Province (No. Y229461211); Science and Technology Plan Projects in Qinghai Province (No. 2015-ZJ-733); and the National Natural Science Foundation of China (No. 81874331).

## Conflict of Interest

The authors declare that the research was conducted in the absence of any commercial or financial relationships that could be construed as a potential conflict of interest.

## Abbreviations

ALT, alanine aminotransferase; AST, aspartate aminotransferase; CD36, cluster of differentiation 36; FAS, fatty acid synthetase; FFA, free fatty acid; GDS, gut-derived serotonin; HTR2A, 5-hydroxytryptamine receptor 2A; IL-6, interleukin 6; LC-QTOF MS, liquid chromatography-hybrid quadrupole time-of-flight mass spectrometry; MCP-1, monocyte chemoattractant protein 1; NAFL, nonalcoholic fatty liver; NAFLD, nonalcoholic fatty liver disease; NAS, NAFLD activity score; NASH, non-alcoholic steatohepatitis; PLIN 2, perilipin 2; PPARγ2, peroxisome proliferator activated receptor gamma 2; TC, total cholesterol; TG, triglyceride; TNFα, tumor necrosis factor α.
