# Supplementary material for: Gut-Derived Serotonin Contributes to the Progression of Non-Alcoholic Steatohepatitis via the Liver HTR2A/PPARγ2 Pathway
Source: Front Pharmacol. 2020 May 14;11:553. doi: 10.3389/fphar.2020.00553 (PMC7240039; doi:10.3389/fphar.2020.00553)
Supplement: Supplementary file 1 [file DataSheet_1.zip › Supplementary Material/Supplementary Material.docx]

Supplementary Material

**Supplementary Figures.**

**FIGURE S1 |** Increased gut-derived serotonin signaling in the duodenum was correlated with non-alcoholic fatty liver disease/non-alcoholic steatohepatitis (NAFLD/NASH). (**A**) The content of 5-HT positive (5-HT^+^) cells in duodenum of SD rats. (**B**) The semi-quantitative values of 5-HT positive (5-HT^+^) cells in duodenum of SD rats. (**C**) The serum serotonin level of SD rats fed HFD diets during 10, 20, 30, 40, 50 days. All data are presented as the mean ± SEM (n=6). ^#^*p* < 0.05, ^##^*p* < 0.01, ^###^*p* < 0.001, compared with corresponding control group.

**FIGURE S2 |** Gut-derived serotonin deficiency ameliorated the progression of non-alcoholic steatohepatitis. The serum lipid level and liver function of SD rats with administration TRP Free diet or LP533401, total triglyceride (TG) (**A**), total cholesterol (TC) (**B**), high density lipoprotein (HDL) (**C**), low density lipoprotein (LDL) (**D**), Alanine aminotransferase (ALT) (**E**), Aspartate aminotransferase (AST) (F). (G-I) The metabolic parameters of SD rats. All data are presented as the mean ± SEM (n=6). ^#^*p* < 0.05, ^##^*p* < 0.01, ^###^*p* < 0.001, compared with corresponding control group; ^*^*p* < 0.05, ^**^*p* < 0.01, ^***^*p* < 0.001, compared with relative model group.

**FIGURE S3 |** Gut-derived serotonin deficiency ameliorated the progression of non-alcoholic steatohepatitis. (**A**) The content of 5-HT^+^ positive cell in liver. (**B**) NAFLD activity score (NAS) of each group of NASH SD rats after treatment. (**C**) Semi-quantitative analysis of the contents of lipid droplets. (**D**) The content of inflammation factors (Tnf-α, Il-1β, Il-6) in the serum of Rats. All data are presented as the mean ± SEM. ^##^*p* < 0.01, ^###^*p* < 0.001, compared with corresponding control group; ^*^*p* < 0.05, ^**^*p* < 0.01, ^***^*p* < 0.001 compared with relative model group.

**FIGURE S4 |** Gut-derived serotonin promoted lipid synthesis and the inflammatory response through the HTR2A receptor in hepatocytes. (**A**) Semi-quantitative analysis of the protein expression of HTR2 subtypes in liver and BRL-3A cells (**B**). All data are presented as the mean ± SEM (n = 6). ^#^*P* < 0.05, ^##^*P* < 0.01, compared with corresponding control group; ^*^*P* < 0.05, ^**^*P* < 0.01, compared with relative model group.

**FIGURE S5 |** HTR2A/PPARγ2 signaling pathway was involved in the lipogenesis and increased the genes expression of inflammation. (**A**) Oil red O staining of BRL-3A (× 400). (**B**) The lipid content of TG in BRL-3A. The mRNA expression of genes related with lipid synthesis (**C**) (Fas, Cd36, Plin2) and inflammation response (**D**) (Tnf-α, Il-6, Mcp-1) in BRL-3A. Semi-quantitative analysis of the protein expression of PPARγ2 in liver (**E**) and BRL-3A (**F**). “C” means control group (1% BSA with FFA free), “M” means 1 mM FFA treatment; “5-HT” means 5-HT (50 μM) treatment; “5-HT + M” means 5-HT (50 μM) with FFA (1 mM) treatment; “M+T” represents FFA(1 mM) with TCB-2 (0.1 μM) treatment; “M+PIO” represents FFA(1 mM) and 5-HT (50 μM) with Pioglitazone (1 μM); “M+GW” represents FFA(1 mM) and 5-HT (50 μM) with GW9662 (10 μM). All data are presented as the mean ± SEM (n=6). ^#^*p* < 0.05, ^##^*p* < 0.01, ^###^*p* < 0.001, compared with corresponding control group; ^*^*p* < 0.05, ^**^*p* < 0.01, ^***^*p* < 0.001 compared with relative model group; ^&&^*p* < 0.01, ^&&&^*p* < 0.001, compared with 5-HT+M group.

**FIGURE S1**


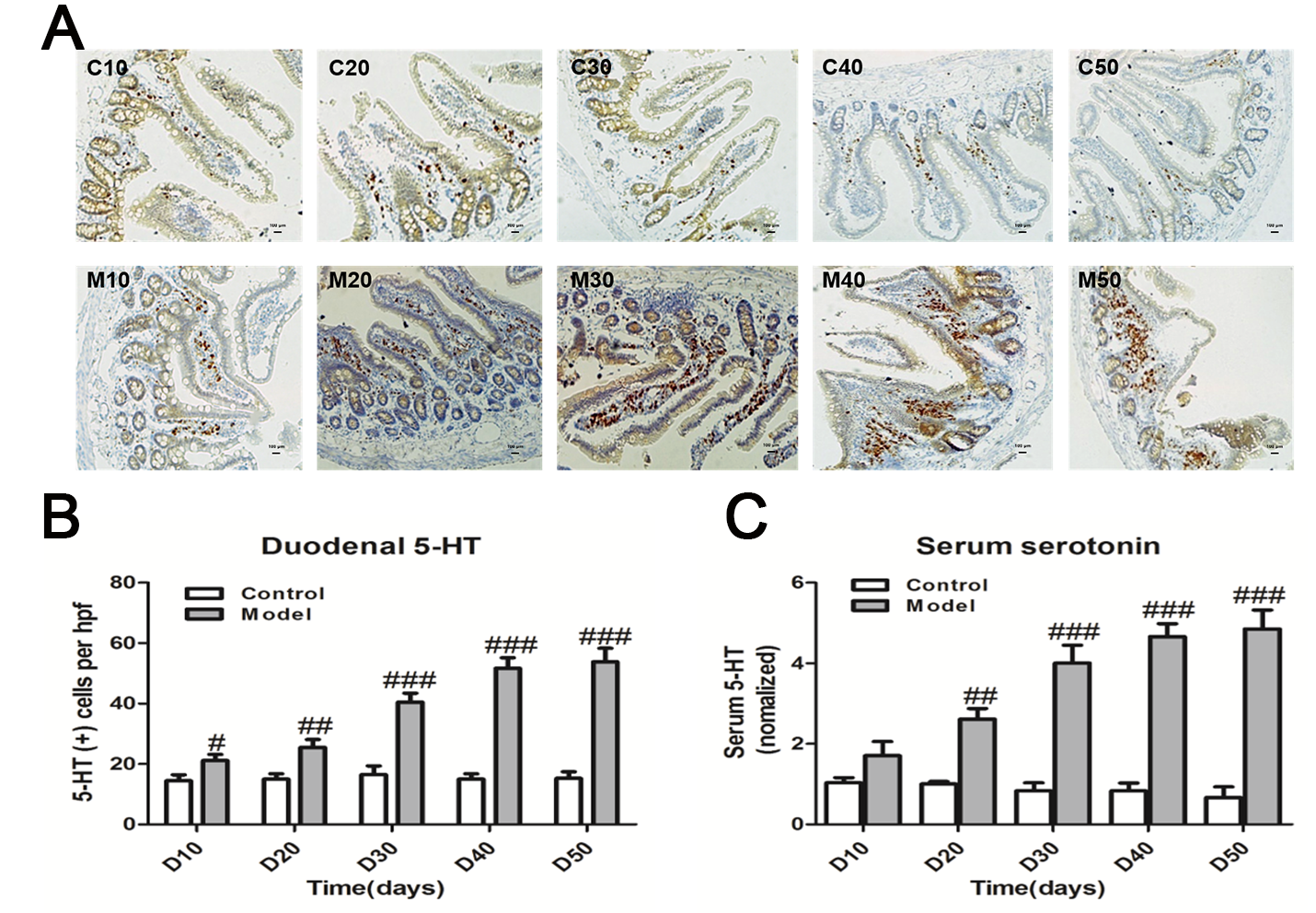


**FIGURE S2**


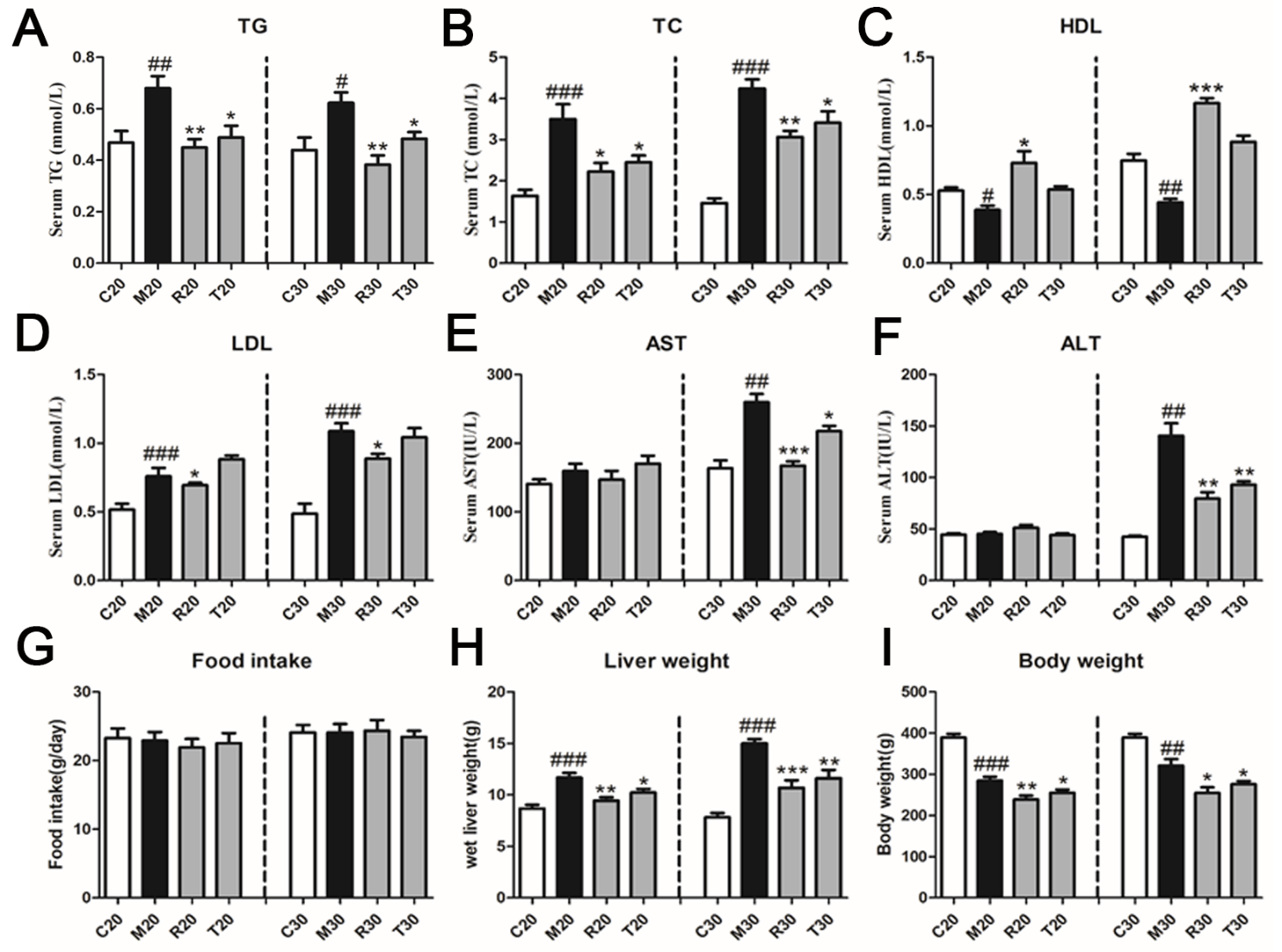


**FIGURE S3**


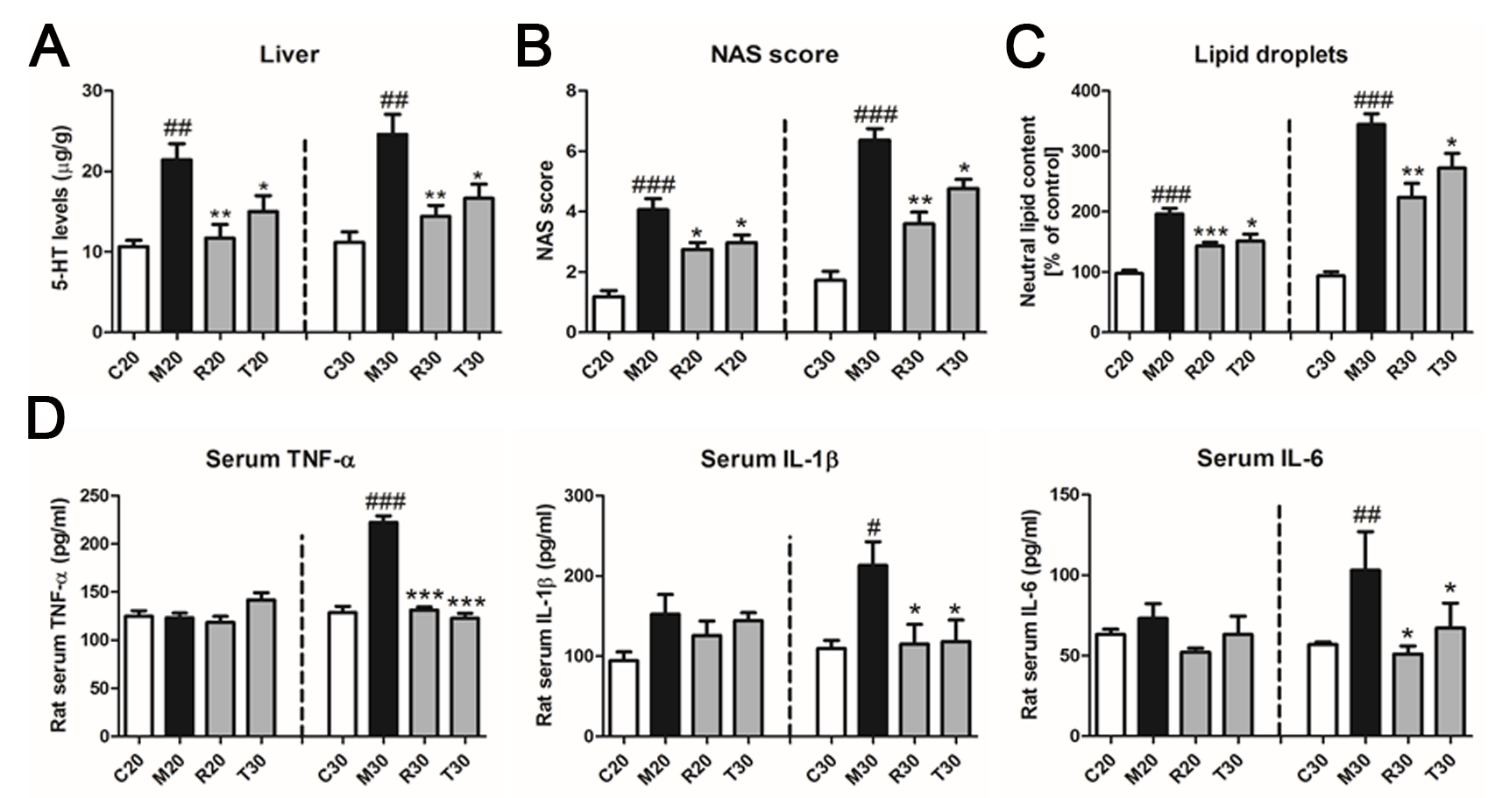


**FIGURE S4**


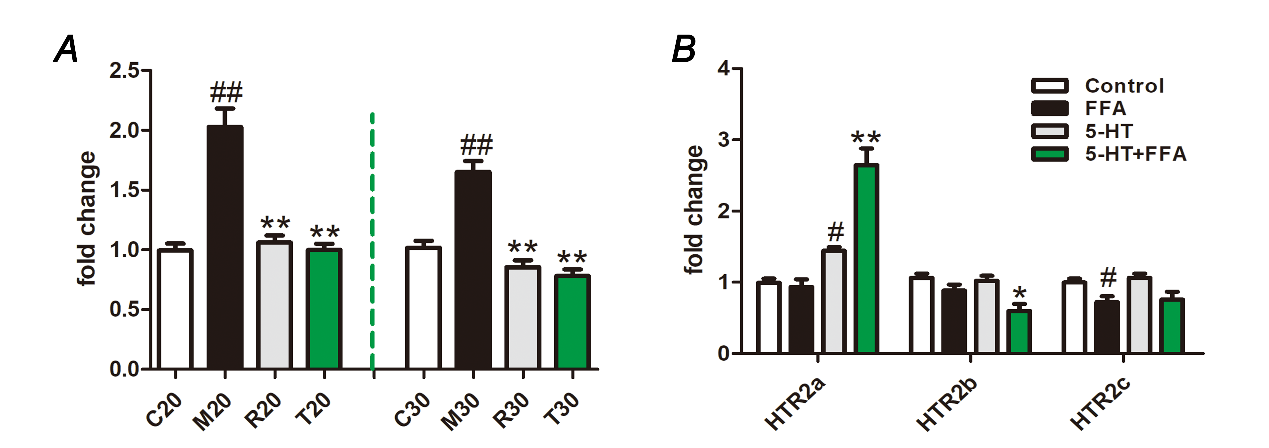


**FIGURE S5**


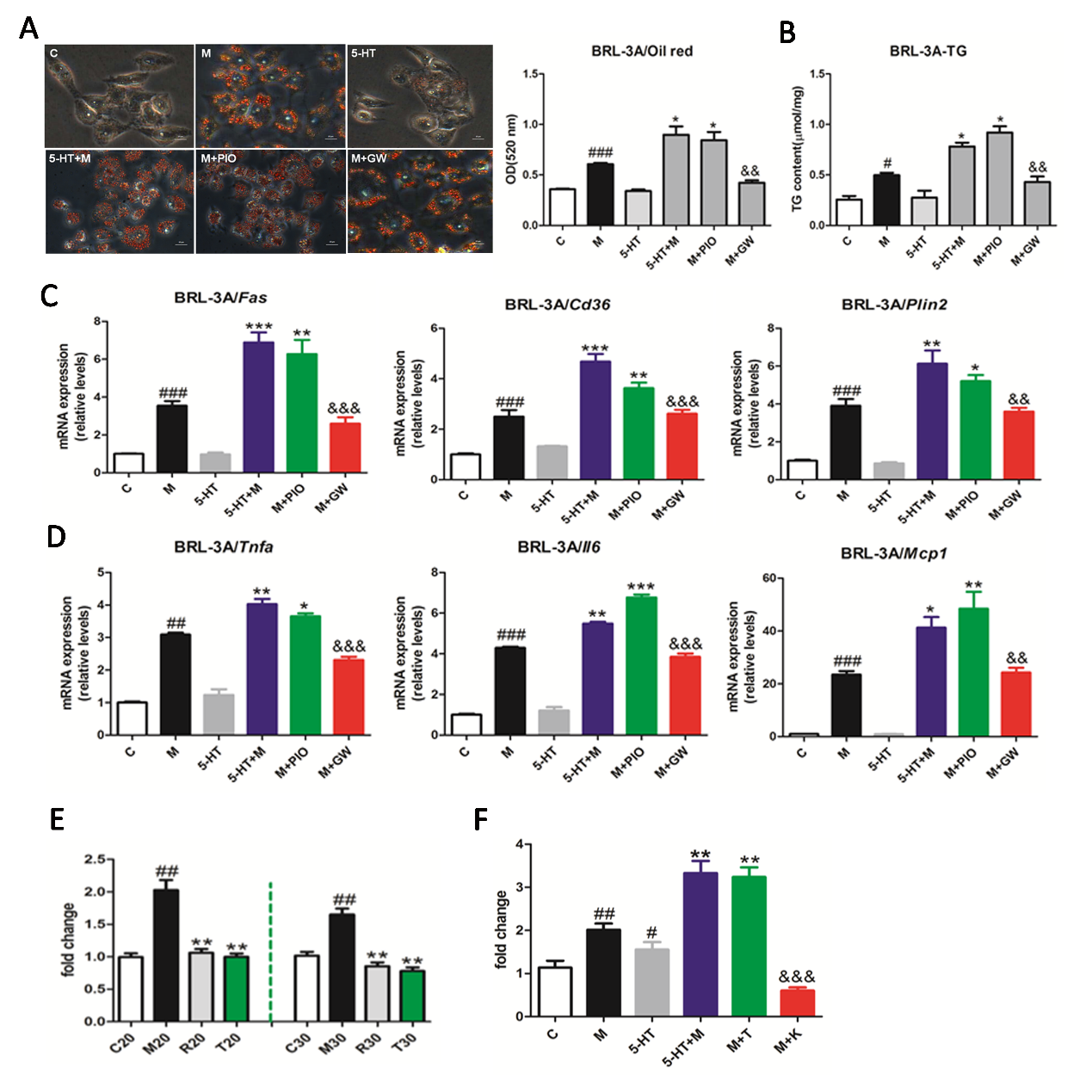


**Supplemental Experimental Procedures**

**Table S1 |** The gradient elution of LC-QTOF MS/MS

| **Time (min)** | **A(V%)** | **B(V%)** |
| --- | --- | --- |
| 0 | 95 | 5 |
| 3 | 95 | 5 |
| 3.1 | 0 | 100 |
| 8 | 0 | 100 |

Mobile phase composition: A: 0.1% aqueous formic acid (ESI^+^), B: acetonitrile.

Column temperature: 30 °C; Flow rate: 0.25 mL/min; Post time: 8 min

**Table S2** **|** The parameter settings of LC-QTOF MS/MS

| **Parameter** | **Value** |
| --- | --- |
| Gas Temp (℃) | 325 |
| Gas Flow (L/min) | 8 |
| NebμLizer (psig) | 40 |
| Sheath Gas Temp (℃) | 350 |
| Sheath Gas Flow (L/min) | 12 |
| V Cap | 4000 |
| Nozzle Voltage (V) | 0 (+) |
| Fragmentor | 150 |
| Skimmer | 65 |
| Octopole RF Peak | 750 |

Ion source: ESI source, positive ion mode; Scanning interval: 50-1000 m/z; Purine (121.050873) and HP-0921 (922.009798) were used as reference ions in the positive ion mode.

**Table S3 |** Primers used for quantitative real-time polymerase chain reaction

| **Gene name**  *Rattus norvegicus* | **Forward primer (5'->3')** | **Reverse primer (5'->3')** | **Accession number** |
| --- | --- | --- | --- |
| Pparα | GCATGGCTGAGAAGACGCTTG | GGATAGCCTTGGCAAATTCCG | [NM_013196.1](http://www.ncbi.nlm.nih.gov/entrez/viewer.fcgi?db=nucleotide&id=6981381) |
| Pparγ1 | GTCAAAGGAATGGGAGTGGTC | CTGTTTTATGCTGTTATGGGTG | NM_001145367.1 |
| Pparγ2 | CAGGTTTGGGCGAATG | TTTGGTCAGCGGGAAG | [NM_001145366.1](http://www.ncbi.nlm.nih.gov/entrez/viewer.fcgi?db=nucleotide&id=223941853) |
| Srebp1c | GGAGCCATGGATTGCACATT | GCTTCCAGAGAGGAGCCCAG | [NM_001276708.1](http://www.ncbi.nlm.nih.gov/entrez/viewer.fcgi?db=nucleotide&id=453040313) |
| Fas | CAACATTGACGCCAGTTCCG | TTCGAGCCAGTGTCTTCCAC | [NM_017332.1](http://www.ncbi.nlm.nih.gov/entrez/viewer.fcgi?db=nucleotide&id=8394157) |
| Cd36 | CCACTCCAGAACCCAGAC | TCCAGCACACCATACGAC | NM_031561.2 |
| Plin 2 | TTGGCTNATGCCCTTTNGTAA | GACCACCATAAAGAGGAGACNAG | NM_001308145.1 |
| Tnfα | CACCATGAGCACGGAAAGCATGA | CGCCTCACAGAGCAATGACTCCA | [NM_012675.3](http://www.ncbi.nlm.nih.gov/entrez/viewer.fcgi?db=nucleotide&id=260166688) |
| Il6 | CACTTCACAAGTCGGAGGCT | AGCACACTAGGTTTGCCGAG | [NM_012589.2](http://www.ncbi.nlm.nih.gov/entrez/viewer.fcgi?db=nucleotide&id=451958166) |
| Mcp1 | GATCCCAATGAGTCGGCTGG | ACAGAAGTGCTTGAGGTGGTT | [NM_031530.1](http://www.ncbi.nlm.nih.gov/entrez/viewer.fcgi?db=nucleotide&id=13928713) |
| Htr1a | GGCTACACCATCTACTCCAC | CCTTCTTTTCCACCTTCC | NM_012585.1 |
| Htr1b | ATGGAGGAGCAGGGTATT | CAGTTGTGGGAGAGGTTG | NM_022225.1 |
| Htr1d | CGAGAAAGGAAAGCCACT | GACCAAGGATACCACAAAGA | NM_012852.1 |
| Htr1f | AACCACCATCAACTCCCT | ATCCAACTCTCGCTCACA | NM_021857.3 |
| Htr2a | ATGTGTTTGTCTGGATTGGT | AGTGGCTTTCTGTTTTCCTT | NM_017254.1 |
| Htr2b | AGCAGAGGAAATGAAGCA | GTGAAACAGCCAGAATAACC | NM_017250.1 |
| Htr2c | GCTATCAACAACGAAAAGAAA | GCCAATCCACACAAACAC | NM_012765.3 |
| Htr3a | GTTCTGGACCGACGAGTT | GCTGTAGGGGCTTGTAGTT | NM_024394.2 |
| Htr3b | GCCACCACTGTCTACCTG | AACTCATCATTCCAAACCTCT | NM_022189.1 |
| Htr4 | GGACAGAGACCAAAGCAG | AAGCCAGAGGAAAGCAGT | NM_012853.1 |
| Htr5a | CCAGACAGAAGGGGACAC | TAACAAAGAAGGGGAACCA | NM_013148.1 |
| Htr5b | ATTCGTTCTTCAATCCCTT | GCCCTGCTTATCTCTGCT | NM_024395.1 |
| Htr6 | CCATCTGCTTCACCTACTG | CTTCCTGCTATGCTTGGT | NM_024365.2 |
| Htr7 | GAGGCAAAATGGGAAATG | AAGAGAGGAGGTAAGGTGATG | NM_022938.2 |
| Tph1 | GTCCCTCTCTTGGCTGAA | TGAACCGTCTCCTCTGAA | NM_001100634.2 |
| Sert | AGCGATGTGAAGGAGATG | TTGTATTGGAAAAGCCGTAG | NM_013034.4 |
| Maoa | AAGACACGCTCAGGAATG | GAACCACAGGGCAGATAC | NM_033653.1 |
| Gapdh | CCTTCATTGACCTCAACTACATGGT | TCATTGTCATACCAGGAAATGAGCT | [NM_017008.4](http://www.ncbi.nlm.nih.gov/entrez/viewer.fcgi?db=nucleotide&id=402691727) |
