# Supplementary figures and images for: Gut-Derived Serotonin Contributes to the Progression of Non-Alcoholic Steatohepatitis via the Liver HTR2A/PPARγ2 Pathway
Source: Front Pharmacol. 2020 May 14;11:553. doi: 10.3389/fphar.2020.00553 (PMC7240039; doi:10.3389/fphar.2020.00553)

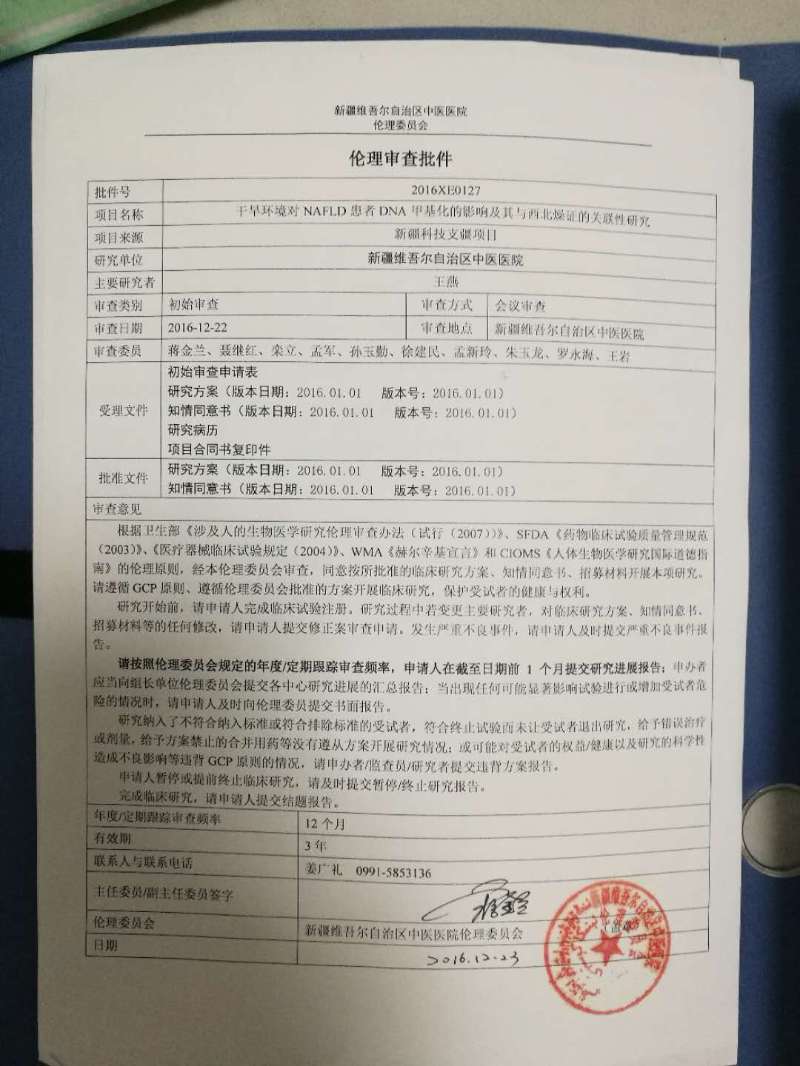

Supplement: Supplementary file 1 [file DataSheet_1.zip › Supplementary Material/Ethical approval.jpg]

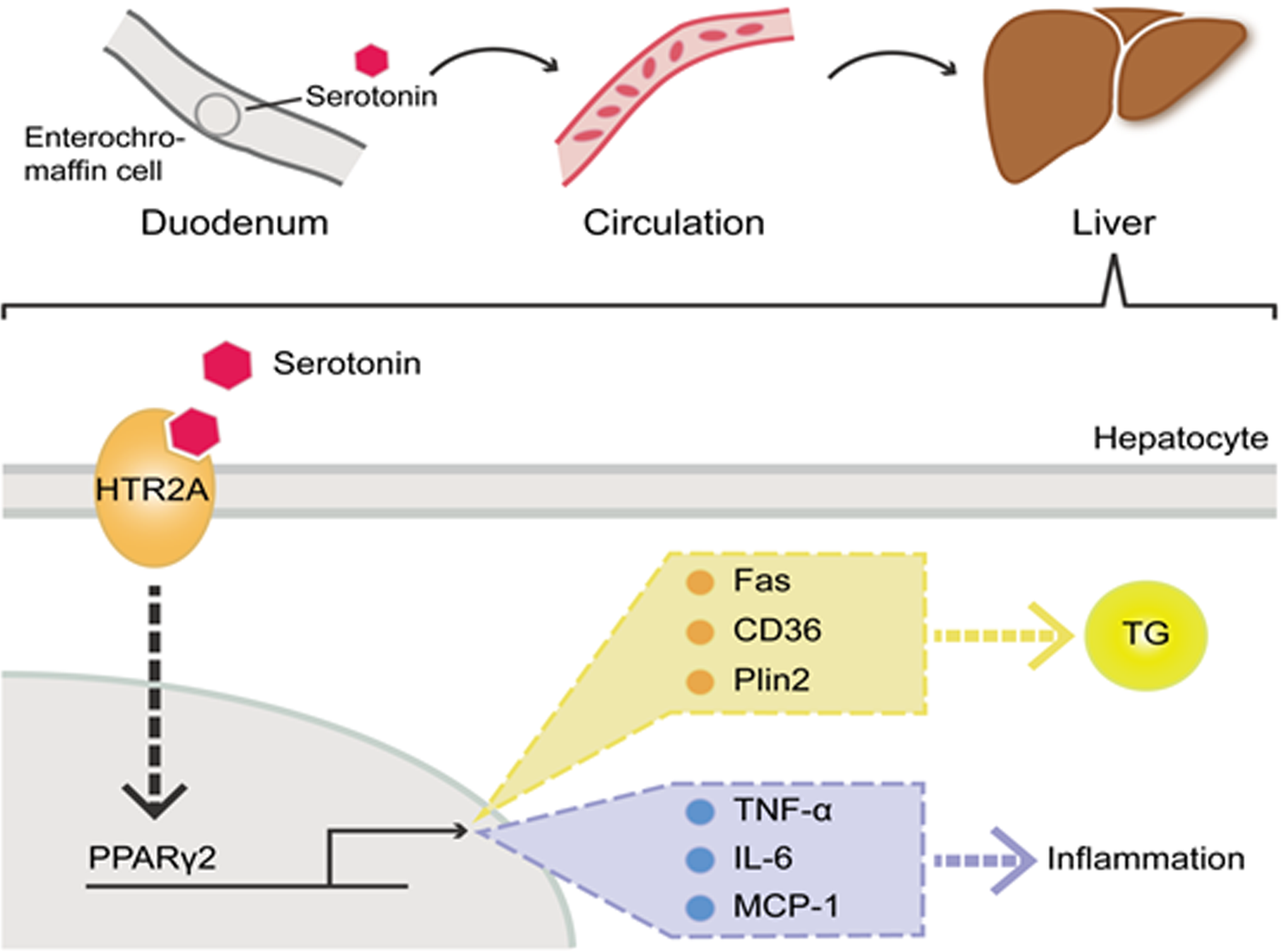

Supplement: Supplementary file 1 [file DataSheet_1.zip › Supplementary Material/Graphical abstract.tif]
